# Novel non-curable resection prediction model for early colorectal cancer following endoscopic submucosal dissection based on inflammatory immune index

**DOI:** 10.3389/fmed.2025.1489842

**Published:** 2025-05-08

**Authors:** Xiunan Li, Lei Zhang, Biao Xu, Shu Ding, Jing Wang, Yu Jia

**Affiliations:** ^1^Digestive Endoscopy Center, Beijing Chaoyang Hospital, Capital Medical University, Beijing, China; ^2^Department of Nursing, Beijing Chaoyang Hospital, Capital Medical University, Beijing, China; ^3^Mass General Cancer Center, Mass General Brigham, Harvard Medical School, Somerville, MA, United States; ^4^Department of Thoracic Surgery, Beijing Institute of Respiratory Medicine and Beijing Chao-Yang Hospital, Capital Medical University, Beijing, China

**Keywords:** ESD-related immune inflammation prediction model ESD, colorectal carcinoma, SII, non-curative resection, nomogram

## Abstract

**Backgrounds:**

Colorectal carcinoma represents one of the common malignant tumors of digestive tract in clinic. Systemic immune inflammation index (SII) has great potential in predicting prognosis of digestive tract tumors. We sought to explore the predictive ability of SII for non-curative resection of early colorectal cancer treated with ESD, and to establish a related predictive model.

**Methods:**

A retrospective analysis was performed on data from patients with early-stage colorectal cancer who underwent ESD in our hospital between January 2019 and December 2022. To establish the optimal cut-off value for the SII, Receiver Operating Characteristic (ROC) curves were generated, correlating preoperative SII levels with postoperative resection outcomes. Patients were categorized into high SII and low SII groups, and their clinical characteristics were comparatively analyzed. Furthermore, patients were stratified according to the presence or absence of non-curative resection outcomes post-ESD, to identify independent risk factors associated with non-curative resection. A prognostic nomogram was subsequently developed to enhance predictive accuracy for non-curative resection, integrating identified risk variables.

**Results:**

A total of 215 patients were enrolled in this study, all of whom successfully underwent ESD, achieving an en bloc resection rate of 96.7%. Based on surgical procedures and pathological resection characteristics, 181 cases were classified as curative resections, whereas 34 cases of non-curative resections. Postoperative complications occurred in 10 patients, resulting in a complication rate of 4.7%. The optimal cut-off value of SII was 629.2 × 10^∧^9/L (area under the curve: 0.762, *P* < 0.001), and the sensitivity and specificity was 64.7 and 85.6%, respectively. An optimal SII cut-off value for predicting non-curative resection was determined to be 1.56 (AUC: 0.571, 95% CI: 0.501–0.641). Multivariate analysis demonstrated that elevated SII (*P* = 0.002), a positive lifting sign (*P* = 0.003), increased tumor size (*P* = 0.034), and poor tumor differentiation (*P* < 0.001) were independent risk factors significantly associated with non-curative resection.

**Conclusion:**

SII revealed well correlation in predicting non-curable resection in patients with early colorectal cancer treated by ESD. Meanwhile, the higher the patient’s NLR, PLR, tumor diameter and infiltration depth, the more likely to occur postoperative non-curative resection.

## Introduction

Colorectal carcinoma represents one of the most common malignant tumors of gastrointestinal malignancies in clinic. According to the latest report published in the CA: A Cancer Journal for Clinicians (1), colorectal cancer ranks as the third most prevalent malignancy worldwide and the second most common cancer in terms of incidence in China. The report further highlights that although the overall incidence and mortality rates of colorectal cancer have been steadily declining, there is a concerning shift towards a younger patient demographic and more advanced disease stages at diagnosis. Additionally, studies suggest that routine colonoscopic screening can reduce the incidence of colorectal cancer by approximately 40% and the mortality rate by around 60%. Data from the U.S. SEER (Surveillance, Epidemiology, and End Results) database indicates that about 37% of colorectal cancer patients are diagnosed at an early stage, with the tumor confined locally, and these patients have a 5-year survival rate of up to 90.9%. In contrast, approximately 22% of patients present with advanced disease, characterized by distant metastasis, and their 5-year survival rate drops significantly to only 15.1%. Therefore, early detection, diagnosis, and intervention are crucial for improving the long-term prognosis of patients with colorectal cancer. In recent years, with the progressive development of endoscopic techniques, endoscopic resection has emerged as a feasible therapeutic option for certain patients with early-stage colorectal cancer, thereby circumventing the morbidity associated with conventional bowel resection surgery. Currently, the most widely adopted method is endoscopic submucosal dissection (ESD). ESD originated as an evolution of endoscopic mucosal resection (EMR) and was first pioneered in 1996 by Japanese researcher Gotoda, who successfully utilized an electrosurgical knife with an insulated ceramic tip to perform en bloc submucosal dissection on early gastric cancer lesions exceeding 2 cm in diameter (2). This landmark advancement catalyzed rapid growth in the application of ESD for the treatment of early gastrointestinal malignancies. Presently, ESD has been established as the standard of care for early-stage mucosal neoplasms of the gastrointestinal tract in Japan (3, 4). However, from an oncological perspective, ESD is not without its limitations, as it carries a risk of non-curative resection, with the incidence reported to be approximately 20% in the literature. In such cases of non-curative resection, subsequent surgical intervention is often warranted to mitigate the risk of local recurrence. Therefore, the development of a robust predictive model to accurately assess the likelihood of non-curative resection during ESD is of paramount importance. Such a model would facilitate the timely formulation of optimal therapeutic strategies and significantly enhance the long-term prognosis of patients.

Tumor-associated inflammation and immune responses are recognized as critical factors in the initiation, progression, angiogenesis, and metastasis of malignancies. Several markers have been proposed to assess these responses, including various inflammation and immune-based scoring systems such as the neutrophil-to-lymphocyte ratio (NLR), platelet-to-lymphocyte ratio (PLR), and monocyte-to-lymphocyte ratio (MLR) (5–7). Recently, the Systemic Immune-Inflammation Index (SII) has been introduced as a novel biomarker derived from the counts of peripheral blood platelets, neutrophils, and lymphocytes. SII has garnered considerable attention in recent years due to its capacity to reflect the host’s inflammatory and immune status. Elevated SII levels have been demonstrated to correlate with poor prognosis in various malignancies, including gastric cancer, hepatocellular carcinoma, and breast cancer. In colorectal cancer, inflammation plays a pivotal role in tumor progression and metastasis, influencing the tumor microenvironment and promoting immune evasion. Elevated SII levels are thought to signify a pro-tumorigenic environment characterized by increased neutrophil-mediated inflammation, thrombocytosis, and lymphocytopenia. These alterations collectively facilitate cancer cell proliferation, invasion, and angiogenesis.

However, research on the prognostic value of the SII in patients with colorectal cancer undergoing ESD remains limited. Given the minimally invasive nature of ESD and the challenges associated with postoperative risk stratification, SII offers a potential non-invasive biomarker for predicting postoperative complications and long-term survival outcomes. Since 2018, our center has employed ESD techniques for the treatment of early-stage gastrointestinal tumors. Through a retrospective analysis of patient data, we aim to investigate the risk factors associated with non-curative resection during ESD and to develop predictive models that focus on the tumor immune-inflammatory status as a primary axis.

## Materials and methods

### Ethics approval and consent to participate

The study was conducted in accordance with the Declaration of Helsinki (as revised in 2013) and approved by the Ethics Committee of Beijing Chaoyang Hospital Affiliated to Capital Medical University (No. 2019-D.-302). Due to the retrospective nature of the study, participant informed consent was waived, and the study design was approved by the appropriate ethics review board.

### Patients and clinicopathological factors

#### Patient selection

**FIGURE 1:**
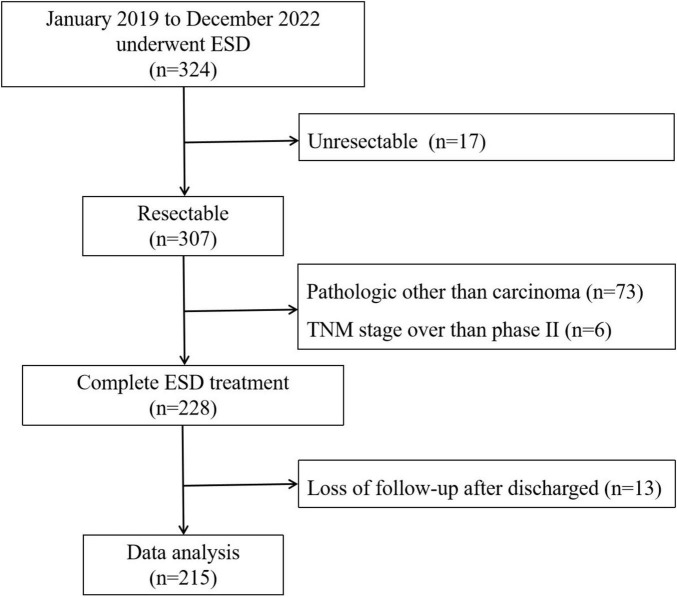
Flowchart of patient selection.

Our study retrospectively analyzed the data of ESD patients in our hospital between January 2019 and December 2022 and screened out 215 eligible patients for further analysis according to the inclusion and exclusion criteria (Figure 1). The authors are accountable for all aspects of the work in ensuring that questions related to the accuracy or integrity of any part of the work are appropriately investigated and resolved.

#### Inclusion and exclusion criteria

Inclusion Criteria: (1) patients with colorectal masses who underwent ESD from January 2018 to December 2022; (2) Patients aged 18 years and above; (3) preoperative evaluation without regional lymph node metastasis and distant metastasis; (4) Intraoperative tumor removal; (5) Postoperative pathological confirmation of colorectal cancer; (6) Postoperative according to the eighth version of AJCC TNM stage belongs to stage I–II patients; (7) The patient’s clinical and follow-up data are complete.

Exclusion criteria: (1) preoperative treatment such as radiotherapy and chemotherapy; (2) The tumor was not removed for various reasons during the operation; (3) Postoperative pathology confirmed as non-colorectal cancer; (4) Incomplete data or loss of follow-up during follow-up.

### Endoscopic submucosal dissection operation

The procedures were performed by the same endoscopist team, routine bowel preparation is performed the day before surgery, water is fasted on the day of surgery, and intravenous anesthesia is given. Surgical operation: (1) Marking: a transparent cap is attached to the end of the inner lens, and after indigo rouge staining determines the scope of the lesion, it is marked with Dual knife ring lesion dot electrocoagulation about 5 mm on the outer edge of the lesion. (2) Submucosal injection: submucosal injection of indigo carmine and hyaluronic acid mixture at the electrocoagulation marker at the outer edge of the ring lesion, 2 mL per place to the lesion uniform bulge. (3) Incision: use a needle-like knife to cut the lesion mucosa at the lesion marker point. (4) Dissection: After cutting the mucosa around the lesion, peel off the lesion with a Dual knife or IT knife along the submucosa to peel off the lesion to a whole piece. (5) Wound treatment: After the lesion is completely peeled off, the small blood vessels exposed by the wound are stopped with hot hemostatic forceps, and if the wound is deep, titanium clamps are given to clamp the mucosa around the wound if necessary. (6) Specimen treatment: mark the peeling surface of the specimen, mark the oral side and side, put 10% neutral formalin into fixation, and make pathological sections for pathological diagnosis through standard processing procedures.

### Patient grouping and study definitions

Patients were stratified based on preoperative peripheral blood parameters. NLR was calculated as the absolute neutrophil count divided by the absolute lymphocyte count. PLR was defined as the platelet count divided by the absolute lymphocyte count. SII was calculated using the formula: absolute neutrophil count multiplied by platelet count divided by the absolute lymphocyte count (10^∧^9/L). To determine the optimal cut-off values for NLR, PLR, and SII, ROC curves were constructed based on the occurrence of non-curative resections postoperatively, and patients were grouped accordingly. According to the 2020 guidelines from the Japan Gastroenterological Endoscopy Society for colorectal EMR/ESD, the nature of endoscopic resection was classified into eCura A, eCura B, and eCura C categories. Curative resections included eCura A and eCura B, while non-curative resections were classified as eCura C (8). Collected data included demographic and clinical characteristics such as age, gender, smoking status, preoperative serum carcinoembryonic antigen (CEA) levels, white blood cell count, neutrophil count, lymphocyte count, platelet count, NLR, PLR, SII, albumin levels, and duration of surgery. Endoscopic characteristics were also recorded, including tumor location, tumor size, gross type, and presence of the lifting sign. Pathological features collected included depth of invasion, lymphatic invasion, perineural invasion, vascular invasion, horizontal and vertical margin status, and tumor differentiation.

### Follow-up strategy

For patients who underwent curative resection, regular postoperative follow-up was conducted. In cases of non-curative resection, patients were advised to undergo additional surgical treatment, followed by regular follow-up. The follow-up schedule included re-evaluation at 1 month and 3 months post-surgery, every 3 months for the first 2 years, and every 6 months thereafter. The primary endpoints of follow-up were tumor recurrence and patient mortality.

### Statistical analysis

Continuous variables following a normal distribution were expressed as mean ± standard deviation, while those not following a normal distribution were presented as median (interquartile range). Comparisons of categorical data between groups were performed using the Chi-square test. If any expected frequency was less than 1, Fisher’s exact test was applied. Binary logistic regression analysis was employed to identify risk factors. A *p*-value of less than 0.05 was considered statistically significant. All statistical analyses were conducted using SPSS software version 24.0.

## Results

### Baseline characteristics and surgical outcomes

A total of 215 patients were enrolled in this study, comprising 110 males and 105 females, with a male-to-female ratio of 1.05:1. The mean age of the cohort was 61.2 ± 10.5 years, and 60 patients had a documented history of smoking. All patients successfully underwent ESD, achieving an en bloc resection rate of 96.7% (208 out of 215). The mean duration of the procedure was 70.0 ± 17.7 min. Tumor locations were distributed as follows: 82 lesions in the right colon, 57 in the left colon (including the sigmoid colon), and 76 in the rectum. Endoscopic morphological classification identified 38 cases of granular laterally spreading tumors (LSTs), 98 cases of non-granular LSTs, and 79 cases of elevated-type lesions. A positive lifting sign was noted in 193 patients. Histopathological evaluation revealed an average tumor size of 18.2 ± 6.0 mm. The depth of tumor invasion included 128 cases confined to the mucosal layer, 81 cases involving the submucosal layer, and 6 cases extending into the muscularis propria. Tumor differentiation status was categorized as well-differentiated in 172 cases and poorly differentiated in 42 cases. Based on operative findings and pathological assessment of resection margins, 181 cases were deemed curative resections (eCura A in 150 cases and eCura B in 31 cases), while 34 cases were classified as non-curative resections (eCura C).

Postoperative complications were observed in 12 patients, resulting in an overall complication rate of 5.6%. The primary complications included gastrointestinal perforation in 4 patients, which were effectively managed with intraoperative metallic clip closure. Postoperative hemorrhage occurred in 6 patients, all of whom responded favorably to pharmacologic hemostasis. Additionally, 3 cases of postoperative intra-abdominal infection were recorded, which were successfully treated with antibiotic therapy.

### Subsequent treatment and follow-up

Among the 181 patients who underwent curative resection, no additional treatment was administered postoperatively. During a median follow-up period of 34 months, there were no cases of tumor recurrence. In contrast, of the 34 patients who underwent non-curative resection, 19 received subsequent surgical intervention. During a median follow-up period of 31 months, one patient experienced tumor recurrence at 17 months and subsequently died at 24 months. Additionally, three other patients experienced recurrence at 11, 16, and 23 months postoperatively, respectively. The remaining patients did not show any signs of tumor recurrence.

### Determination of optimal cut-off values for inflammatory markers

**FIGURE 2:**
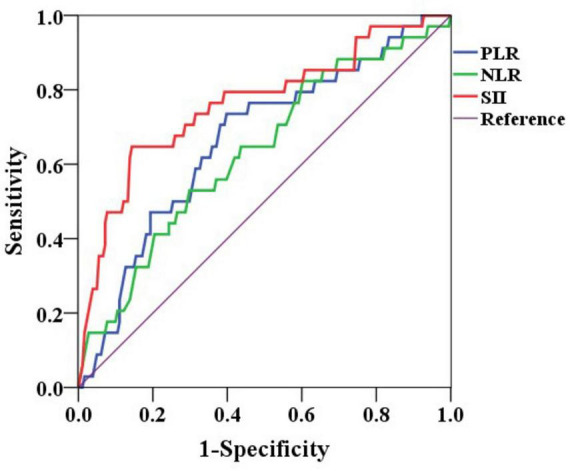
ROC curves of NLR, PLR, and SII for predicting non-curative resection. NLR, neutrophil-to-lymphocyte ratio; PLR, platelet-to-lymphocyte ratio; SII, systemic immune inflammation index.

ROC curves were constructed based on the presence of non-curative resection to determine the optimal cut-off values for inflammatory markers (Figure 2). The optimal cut-off value for the NLR was identified as 2.2 (AUC: 0.633, *P* = 0.014, 95% CI: 0.529–0.736), with a sensitivity of 52.9% and a specificity of 70.2% for predicting non-curative resection. For the PLR, the optimal cut-off value was 109.7 (AUC: 0.666, *P* = 0.002, 95% CI: 0.567–0.764), which provided a sensitivity of 73.5% and a specificity of 60.2% for predicting non-curative resection. The SII had the best performance, with an optimal cut-off value of 629.2 × 10^∧^9/L (AUC: 0.762, *P* < 0.001, 95% CI: 0.665–0.858), yielding a sensitivity of 64.7% and a specificity of 85.6%. The SII was the most effective marker for predicting non-curative resection outcomes.

### Comparison of patients in different systemic immune inflammation groups

Based on the optimal cut-off value of 629.2 × 10^∧^9/L for SII, patients were divided into high SII and low SII groups. A comparison of clinical data between the two groups is presented in Table 1. The results indicated significant differences between the two groups in terms of NLR, PLR, tumor size, tumor invasion depth, and nature of resection (*P* < 0.05). Patients with higher SII values tended to have higher NLR and PLR, larger tumor diameters, deeper invasion depths, and were more likely to undergo non-curative resection postoperatively.

**TABLE 1:** Comparison of clinical characteristics between patients in different SII groups.

Item	High SII group (*n* = 48)	Low SII group (*n* = 167)	*P*
Age (y)			0.443
≤60	18	73	
>60	30	94	
Gender			0.146
Male	29	81	
Female	19	86	
Smoke			0.188
Yes	17	43	
No	31	124	
CEA (ng/mL)			0.778
≤5	38	129	
>5	10	38	
White blood cell count (10^∧^9/L)			0.061
≤9.5	40	156	
>9.5	8	11	
Neutrophil count (10^∧^9/L)			0.323
≤6.3	41	151	
>6.3	7	16	
Lymphocyte count (10^∧^9/L)			0.120
≤3.2	48	155	
>3.2	0	12	
Platelet count (10^∧^9/L)			0.115
≤350	42	159	
>350	6	8	
NLR			0.013
≤2.2	10	131	
>2.2	38	36	
PLR			<0.001
≤109.7	15	103	
>109.7	33	64	
Albumin (g/L)			0.277
≤40	38	119	
>40	10	48	
Duration of ESD (min)			0.092
≤60	19	45	
>60	29	122	
Tumor location			0.662
>Right colon	18	64	
Left colon (including sigmoid)	15	42	
Rectum	15	61	
Tumor size (cm)			<0.001
≤3	33	151	
>3	15	16	
Gross type			0.809
Elevated type	17	62	
Granular laterally spreading type	10	28	
Non- granular laterally spreading type	21	77	
Lifting sign			0.076
>Positive	37	146	
Negative	11	21	
Depth of invasion			0.029
Mucosal layer	26	102	
Submucosal layer	18	63	
Muscularis layer	4	2	
Tumor differentiation			0.515
Differentiated	38	139	
Undifferentiated	10	28	
Resection type			<0.001
Curative resection	26	155	
Non- curative resection	22	12	
Complications			0.898
Yes	3	9	
No	45	158	

CEA, carcinoembryonic antigen; NLR, neutrophil-to-lymphocyte ratio; PLR, platelet-to-lymphocyte ratio; SII, systemic immune inflammation index.

### Risk factors analysis for non-curative resection

Based on postoperative assessment, all patients were categorized into a curative resection group (181 cases, 84.2%) and a non-curative resection group (34 cases, 15.8%). Univariate analysis (Table 2) indicated that preoperative white blood cell count, NLR, PLR, SII, tumor size, lifting sign, invasion depth, and tumor differentiation were potential risk factors associated with non-curative resection. Further multivariate analysis (Table 3) revealed that SII (*P* = 0.002, 95% CI = 2.228–30.586), positive lifting sign (*P* = 0.003, 95% CI = 1.885–23.564), tumor size (*P* = 0.034, 95% CI = 1.102–11.762), and tumor differentiation (*P* < 0.001, 95% CI = 4.057–53.662) were independent risk factors for non-curative resection.

**TABLE 2:** Univariate analysis of risk factors for non-curative resection.

Item	Non-curative resection group (*n* = 34)	Curative resection group (*n* = 181)	*P*
Age (y)			0.883
≤60	14	77	
>60	20	104	
Gender			0.100
Male	13	97	
Female	21	84	
Smoke			0.146
Yes	6	54	
No	28	127	
CEA (ng/mL)			0.245
≤5	29	138	
>5	5	43	
White blood cell count (10^∧^9/L)			0.021
≤9.5	27	169	
>9.5	7	12	
Neutrophil count (10^∧^9/L)			0.083
≤6.3	27	165	
>6.3	7	16	
Lymphocyte count (10^∧^9/L)			0.255
≤3.2	34	169	
>3.2	0	12	
Platelet count (10^∧^9/L)			0.330
≤350	30	171	
>350	4	10	
NLR			0.013
≤2.2	16	125	
>2.2	18	56	
PLR			<0.001
≤109.7	9	109	
>109.7	25	72	
SII (10^9^/L)			<0.001
≤629.2	12	155	
>629.2	22	26	
Albumin (g/L)			0.727
≤40	24	133	
>40	10	48	
Duration of ESD (min)			0.961
≤60	10	54	
>60	24	127	
Tumor location			0.730
Right colon	14	68	
Left colon (including sigmoid)	10	47	
Rectum	10	66	
Tumor size (cm)			<0.001
≤3	22	164	
>3	12	17	
Gross type			0.824
Elevated type	14	65	
Granular laterally spreading type	6	32	
Non- granular laterally spreading type	14	84	
Lifting sign			0.001
Positive	22	161	
Negative	12	20	
Depth of invasion			<0.001
Mucosal layer	11	117	
Submucosal layer	18	63	
Muscularis layer	5	1	
Tumor differentiation			<0.001
Differentiated	19	158	
Undifferentiated	15	23	

CEA, carcinoembryonic antigen; NLR, neutrophil-to-lymphocyte ratio; PLR, platelet-to-lymphocyte ratio; SII, systemic immune inflammation index.

**TABLE 3:** Multivariate analysis of risk factors for non-curative resection.

Item	*B*-value	OR value	*p*-value	95% CI
Preoperative WBC	0.825	2.282	0.301	0.478–10.902
NLR	−0.210	0.810	0.751	0.221–2.973
PLR	0.934	2.546	0.088	0.869–7.454
SII	2.111	8.255	0.002	2.228–30.586
Tumor size	1.281	3.600	0.034	1.102–11.762
Lifting sign	1.897	6.665	0.003	1.885–23.564
Depth of invasion	0.872	2.391	0.063	0.954–5.992
Tumor differentiation	2.692	14.755	<0.001	4.057–53.662

NLR, neutrophil-to-lymphocyte ratio; PLR, platelet-to-lymphocyte ratio; SII, systemic immune inflammation index.

### Development of a nomogram model for non-curative resection

**FIGURE 3:**
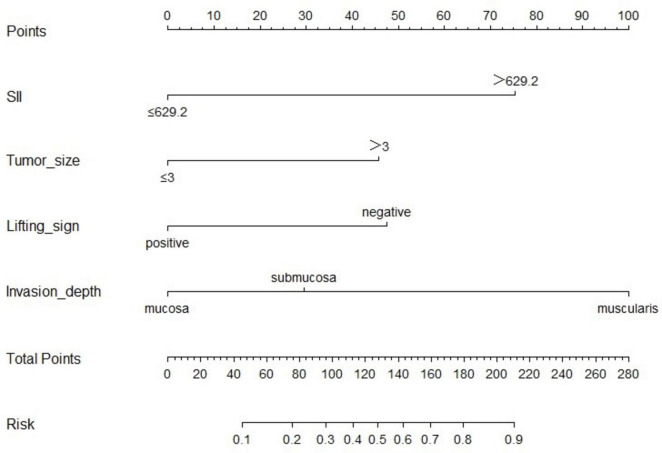
Nomogram for predicting non-curative resection in early colorectal cancer treated with ESD.

Based on the results of the logistic regression analysis, a predictive model was established to assess the likelihood of non-curative resection postoperatively (Figure 3). The model assigns scores to the following factors: SII > 629.2 (75 points), tumor size > 3 cm (46 points), negative lifting sign (48 points), muscularis propria invasion (100 points), and submucosal invasion (30 points). The total score is calculated by summing the points for each risk factor. This total score corresponds to the model’s estimated risk of non-curative resection, with the overall score ranging from 0 to 269 points.

## Discussion

Over the past two decades, Endoscopic Submucosal Dissection has increasingly demonstrated its distinct advantages for the resection of early gastrointestinal cancers. By ensuring therapeutic efficacy while adhering to the principles of minimally invasive surgery, ESD allows patients to avoid more extensive surgical resections. Currently, the application of ESD in the treatment of early-stage colorectal cancer has gained widespread acceptance (9, 10). According to the literature, compared to Endoscopic Mucosal Resection, ESD achieves an en bloc resection rate of approximately 90% for early colorectal cancer (11, 12). However, during clinical practice, it is imperative to rigorously select appropriate indications for ESD to reduce the incidence of non-curative resections. Presently, it is generally recognized that adenomas and intramucosal carcinomas constitute absolute indications for ESD, whereas submucosal carcinoma is considered a relative indication (13, 14). In a multicenter retrospective study conducted by Spadaccini et al., the non-curative resection rate for early colorectal submucosal carcinoma managed with ESD was reported to be 34.3% (207 out of 604 cases), with 126 of these patients subsequently undergoing additional surgical intervention following non-curative resection (15). Although our study corroborates that the depth of tumor invasion serves as an independent risk factor for non-curative resection, accurately assessing invasion depth preoperatively remains a significant challenge. This highlights the necessity for a reliable, efficient, and practical assessment method that can be employed to predict the likelihood of non-curative resection beforehand.

Various researches have demonstrated that inflammation plays a critical role in carcinogenesis, tumor invasion, and metastasis. Several inflammatory markers, such as the NLR and PLR, have been shown to possess substantial prognostic value across various cancer types. The SII, an inflammation marker based on platelet, neutrophil, and lymphocyte counts, has proven to be more accurate in predicting the prognosis of gastrointestinal malignancies compared to other inflammatory markers (16–18). Elevated SII values, often resulting from increased neutrophil and platelet counts alongside reduced lymphocyte levels, typically indicate an enhanced inflammatory response and a diminished immune response in the patient. Therefore, elucidating the roles of neutrophils, platelets, and lymphocytes in cancer development and progression could further clarify the relationship between SII and clinical outcomes. Neutrophils can modify the tumor microenvironment through extracellular mechanisms and secrete various inflammatory mediators that promote tumor cell proliferation, invasion, and metastasis to lymph nodes or distant organs via intrinsic pathways. Platelets interact with tumor cells and facilitate their survival and metastasis through multiple mechanisms: they can protect circulating tumor cells from shear stress during circulation, induce epithelial-mesenchymal transition in circulating tumor cells, and enhance tumor cell extravasation to metastatic sites. Lymphocytes are pivotal in mediating the host’s immune response against malignancies, playing a crucial role in tumor defense by inducing cytotoxic cells and inhibiting tumor cell proliferation and migration. Consequently, SII can reflect the body’s tumor burden and potential for metastasis. Professor Hu and his team initially introduced the SII as a biomarker in hepatocellular carcinoma, where its elevation is primarily associated with increased neutrophil and platelet counts and decreased lymphocyte numbers (19). Multiple clinical cohorts have validated the significant prognostic value of SII in various cancers, including pancreatic and lung cancers (17, 20, 21). Our study also indicates that higher SII values correlate with larger tumor diameters and greater invasion depth, potentially aiding in the early prediction of non-curative resection.

The relationship between tumor diameter and the incidence of non-curative resection during ESD is a pivotal issue in clinical practice that warrants careful consideration. Extensive research has consistently demonstrated that an increased tumor diameter is significantly associated with a heightened risk of non-curative resection in ESD. This correlation is likely intricately linked to the pathological features of the tumor, including the depth of invasion and the potential for lymphatic metastasis. In a study by Sato et al., the influence of tumor size on the technical outcomes of ESD for colorectal neoplasms was thoroughly examined. Their findings indicated a markedly elevated incidence of non-curative resection when the tumor diameter exceeded 40 mm. This increase is likely due to the higher probability of submucosal invasion and deep fibrosis in larger tumors, which are factors that restrict the intraoperative field of vision, elevate the technical complexity of the procedure, and alter pathological characteristics, thereby complicating the achievement of complete resection (22).

In parallel, research conducted by Saito et al. demonstrated that larger tumors frequently exhibit irregular margins and more extensive submucosal involvement, characteristics that increase the likelihood of incomplete resection during ESD and lead to non-curative pathological outcomes (23). These findings underscore the necessity for rigorous preoperative assessment and planning to mitigate the risks associated with resecting larger colorectal lesions.

Further studies have shown that tumor diameter is not only associated with the technical complexity of resection but may also influence the biological behavior of the tumor. Patients with larger colorectal cancer tumors are more likely to have occult lymph node metastasis, even if it is not detectable during endoscopy. Lymph node metastasis is a critical factor affecting the prognosis of colorectal cancer, and a larger tumor diameter often indicates a higher potential for lymphatic spread. Consequently, even when preoperative assessments do not reveal lymph node involvement, patients with larger tumors still exhibit a higher incidence of non-curative resection postoperatively. For instance, Hajibandeh et al. reported that larger colorectal cancer tumors are more likely to be associated with higher tumor grade and lymph node metastasis, both of which increase tumor aggressiveness and, thereby, the likelihood of non-curative resection (24). Moreover, larger tumors typically exhibit more extensive angiogenesis and neural invasion, which elevate the risk of intraoperative bleeding and postoperative complications, consequently impacting the success rate of curative resection. Our study also corroborates that larger tumor diameter and deeper invasion depth significantly increase the risk of non-curative resection following ESD. To address the challenges posed by large-diameter tumors, clinicians should conduct comprehensive preoperative evaluations, including the use of high-resolution imaging modalities such as contrast-enhanced CT or MRI to assess tumor invasion depth, boundary clarity, and potential involvement of surrounding tissues. Combining these imaging techniques with tumor marker analysis and pathological examination can provide a more accurate assessment of the tumor’s biological behavior and potential prognosis. For colorectal cancer tumors of significant size, multidisciplinary team (MDT) collaboration involving endoscopy, surgery, and radiotherapy may be considered to reduce the incidence of non-curative resections.

Complications are pivotal metrics for evaluating the safety of ESD, with gastrointestinal perforation being one of the primary complications associated with colorectal ESD. Factors such as the relatively thin submucosal layer of the intestinal wall and restricted intraoperative visibility contribute to an increased risk of perforation during the procedure. A retrospective study by Iwatsubo et al. (25) demonstrated that the incidence of gastrointestinal perforation in ESD for colorectal cancer is approximately 7.1%. In this study, all 36 patients with confirmed intraoperative perforations were successfully treated using endoscopic clipping during the procedure. Previous literature has shown that the occurrence of gastrointestinal perforation is correlated with the size of the lesion and the extent of fibrosis (26, 27). In our current study, the overall incidence of postoperative complications was 5.6%, with a perforation rate of 1.9%, all of which were effectively managed with intraoperative clipping.

Despite the insights provided, this study has several limitations. Firstly, being a single-center retrospective study, there is an inherent risk of selection bias. Secondly, due to its retrospective design, certain endoscopic characteristics of the lesions, such as mucosal folding and fibrosis, were not included as study variables, limiting the ability to establish a clear association with non-curative resection outcomes. Thirdly, the nomogram model proposed in this study requires validation through prospective, multicenter research to confirm its generalizability and clinical utility.

## Conclusion

In conclusion, SII can serve as a valuable predictor for assessing the likelihood of non-curative resection in patients with early-stage colorectal cancer undergoing ESD, demonstrating a strong correlation with surgical outcomes. Higher SII values are associated with increased NLR, elevated PLR, larger tumor diameter, and greater depth of invasion, all of which contribute to a higher risk of non-curative resection postoperatively.

## Data Availability

The raw data supporting the conclusions of this article will be made available by the authors, without undue reservation.
